# Friction in Myocardial Anoxia Leads to Negative Excess Entropy Production, Self-Organization, and Dissipative Structures

**DOI:** 10.3390/ijms23136967

**Published:** 2022-06-23

**Authors:** Yves Lecarpentier, Victor Claes, Jean-Louis Hébert, Xénophon Krokidis, Olivier Schussler, Alexandre Vallée

**Affiliations:** 1 Centre de Recherche Clinique, Grand Hôpital de l’Est Francilien, 77100 Meaux, France; xkrokidis@gmail.com; 2 Department of Pharmaceutical Sciences, University of Antwerp, 2180 Wilrijk, Belgium; victor.claes@scarlet.be; 3 Institut de Cardiologie, Hôpital de la Pitié-Salpêtrière, Assistance Publique-Hôpitaux de Paris, 75013 Paris, France; jean.l.hebert@gmail.com; 4 Département de Chirurgie Thoracique, Hôpital Cochin, Hôpitaux Universitaires Paris Centre, Paris-Descartes Université, Assistance Publique-Hôpitaux de Paris, 75014 Paris, France; olivier.schussler@gmail.com; 5 Department of Epidemiology-Data-Biostatistics, Delegation of Clinical Research and Innovation, Foch Hospital, 92150 Suresnes, France; alexandre.g.vallee@gmail.com

**Keywords:** friction, myocardium, anoxia, far-from-equilibrium thermodynamics, excess entropy production, self-organisation, dissipative structures

## Abstract

Contraction of the heart is caused by actin filaments sliding along myosin filaments. This generates a frictional force inducing wear of the contractile apparatus. We postulated that this process could be exacerbated when the heart was submitted to severe anoxia. Anoxia induced dramatic abnormalities in the molecular properties of actin-myosin crossbridges. We applied the formalism of far-from-equilibrium thermodynamics to the left ventricular papillary muscles (LVPMs) of mammalian rat hearts which had been subjected to a prolonged anoxia (3 h). We showed that when subjected to prolonged anoxia, the heart operated far-from-equilibrium as evidenced by the non-linearity between thermodynamic force (F/T: Frictional force/Kelvin temperature) and thermodynamic flow (v0: myofilament sliding velocity). The rate of entropy production (EPR) was the product of (F/T) and v0. The excess entropy production (EEP) was equal to $\frac{\partial \delta^{2}S}{\partial t}$ = $\frac{\partial F}{T}\delta$vo; (S: entropy). The tribological system remained stable when EEP was positive and became unstable when EEP became negative, thus characterizing instability of the system and reflecting the occurrence of self-organization and possibly dissipative structures. After 3 h anoxia, re-oxygenation induced significant reversibility. About 20% of the myosin heads did not recover despite re-oxygenation. These results may be of importance in the context of heart transplantation where the delay between the time of sampling from the donor and the time of the graft installation in the recipient should be as short as possible.

## 1. Introduction

**Figure 1:**
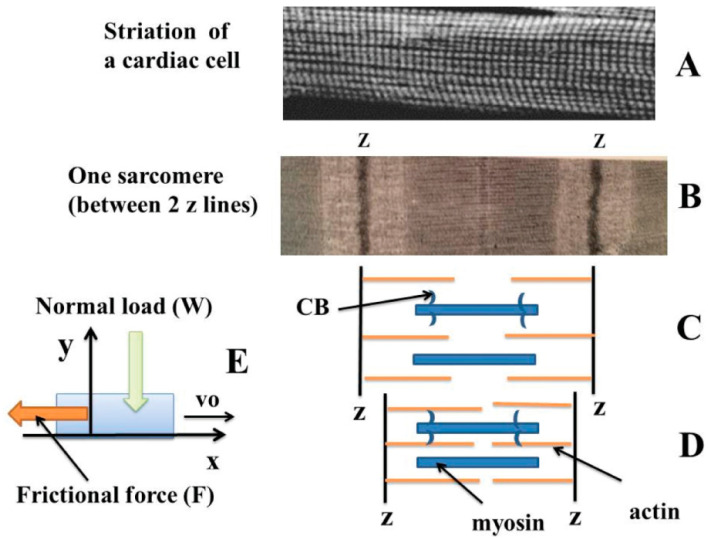
Schematic representations of myofilaments in sarcomeric heart muscle and tribological cardiac system. Cardiomyocytes exhibit a periodic striation characterized by alternating dark A-bands (anisotropic) and light I-bands (isotropic) (**A**,**B**). The central part of the I-band is marked by the Z line. A sarcomere is the smallest functional unit of a striated muscle. It is the repeating unit between two Z-lines (**B**). The I-band is the zone of thin filaments that is not superimposed with thick filaments (myosin). Thin myofilaments are mainly composed of F actin, resulting from the polymerization of many globular actin molecules (G actin). The actin myofilaments are attached by their caudal end to the Z lines. Actin filaments are the major component of the I-band and extend into the A-band. Thick myosin filaments are made up of 200 to 300 molecules of native myosin. Myosin has a long, fibrous tail and a globular head that binds to actin. Myosin filaments are bipolar and extend throughout the A-band. The myosin head also binds to ATP. Muscle contraction results in the sliding of actin filaments along myosin filaments, which is visible only at the level of the I-bands, while the dark A-bands keep a constant length (**C**,**D**) [1,2]. This movement is controlled by the heads of the myosin molecules (CBs), which bind and then detach from the actin molecule. The displacement of myosin on actin is possible through the hydrolysis of ATP molecules. (**E**) refers to the normal (y) and tangential (x) degrees of freedom during dynamic friction. The thermodynamic force was equal to μ W/T, with μ the frictional coefficient, and W the normal load.

The understanding of the mechanical behavior of striated muscles has been considerably improved after the publication of two pioneering articles which laid the foundations for the theory of sliding filaments [1,2]. In fact, actin filaments slide along myosin filaments, resulting in the Z striations coming together and therefore resulting in shortening of sarcomeres (Figure 1A,B). Myosin heads, or crossbridges (CBs), alternatively hook into and unhook from actin filaments. During heart contraction, the sliding of actin and myosin filaments against each other generates friction. A. Huxley also proposed a theoretical formalism to determine CB molecular properties such as the CB attachment and detachment rate constants, as well as the number of active CBs per volume unit, myofilament sliding velocity, and myosin content [3]. The aim of our study was to assess the consequences of a prolonged cardiac anoxia from a thermodynamic point of view by applying the physical concepts established in tribology, the science of interacting surfaces in relative motion.

Ilya Prigogine and colleagues have developed the concept of self-organization to describe complex thermodynamic processes operating far-from-equilibrium [4,5,6,7,8,9,10]. Thus, amplified small fluctuations can spontaneously create patterning, orderliness, self-organization, and possibly dissipative structures. The occurrence of self-organization supposes an open system exchanging energy and matter as well as a non-linear and far-from-equilibrium thermodynamic behavior. Friction and wear are usually considered to generate irreversible processes which lead to energy dissipation and material deterioration. Klamecky introduced the concept of non-equilibrium thermodynamics to describe friction and wear [11]. Barshefsky conducted the first investigations on friction-induced self-organization [12]. Nosonovsky and colleagues developed the thermodynamic principles of irreversible processes used to investigate the formation of spatial and temporal structures induced by friction [13,14]. At the interface, orderliness can increase so that entropy decreases [15,16]. According to Prigogine and colleagues [4,8,10], far-from-equilibrium processes can be a source of order and lead to a new state of matter called dissipative structures. During these processes, the entropy production rate (EPR) can decrease and be followed by the occurrence of a self-organisation process. Due to self-organization, dissipative structures result in reducing EPR, leading to a decreased rate of wear [17,18]. There is no simple criterion to determine if self-organization can occur. However, there is a key condition: self-organization can begin only if the tribosystem has lost its thermodynamic stability, which is characterized by the fact that the excess entropy production (EEP) becomes negative [19]. This is a pre-requisite for self-organization. During friction, self-organization leads to a decrease in wear rate and an increase in the durability of materials.

Huxley’s formalism [3] allowed us to calculate the sliding rate of myofilament (vo) (i.e., the thermodynamic flow) and the thermodynamic frictional force (F/T; F: frictional force and T: Kelvin temperature) generated by the heart. We calculated EPR and EEP and sought to determine whether EEP could become negative after a prolonged period of cardiac anoxia. Cardiac ischemia, the most severe form of which is myocardial infarction, is a leading cause of death worldwide. Despite an encouraging decline, coronary heart mortality remains a major health burden, especially among the elderly [20,21]. Moreover, numerous heart transplants are performed each year worldwide [22]. Our study offers valuable insights for furthering our understanding of the thermodynamic aspects of hypoxic heart diseases and could be of interest in the context of heart transplants where the recipient must benefit from the donor’s heart in the shortest possible time. The main aim of our study was to use far-from-equilibrium thermodynamics to determine whether a cardiac tribosystem submitted to a prolonged anoxia might result in a beneficial self-organization when submitted to dramatic anoxic conditions.

## 2. Results

### 2.1. Mechanical Properties of Left Ventricular Papillary Muscles (LVPMs) and Molecular Myosin CB Characteristics

**Figure 2:**
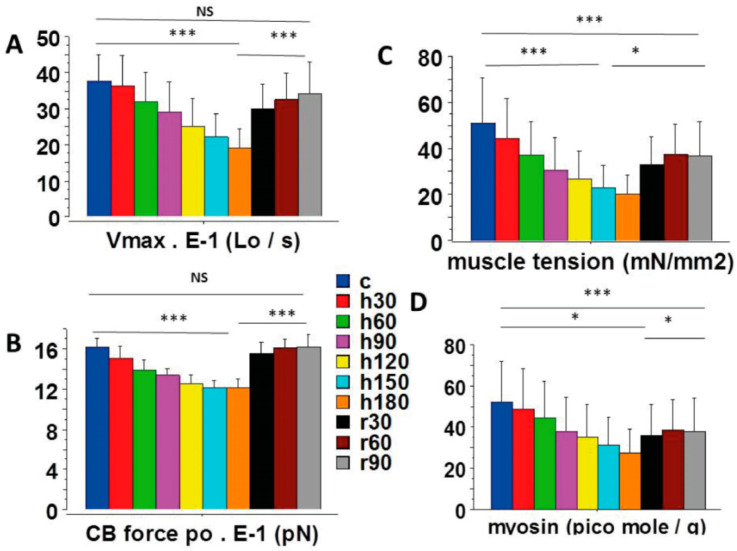
Mechanical parameters of LVPMs and CBs. (**A**): Maximum unloaded shortening velocity (Vmax); (**B**): CB single force (po); (**C**): total muscle tension; (**D**): myosin content. *: *p* < 0.05; ***: *p* < 0.001.

**Figure 3:**
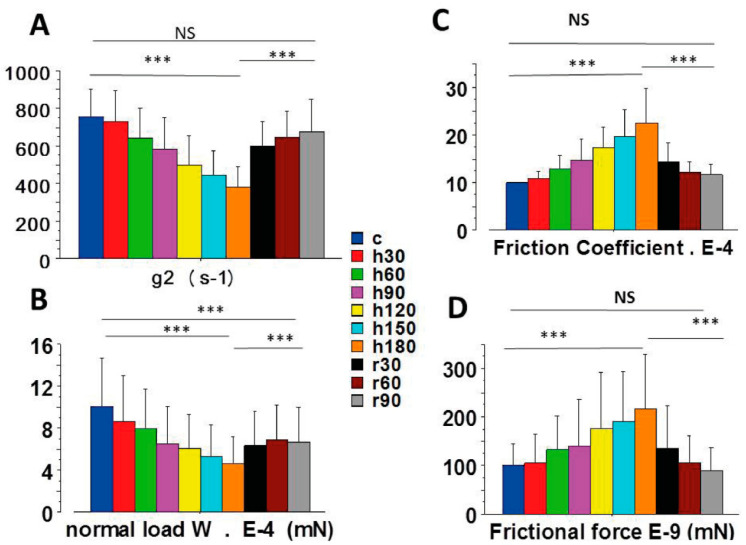
Molecular parameters of CBs. (**A**): CB detachment rate constant (g2); (**B**): normal load (W); (**C**): friction coefficient (μ); (**D**): frictional force (F). ***: *p* < 0.001.

Some mechanical parameters significantly decreased during prolonged cardiac anoxia. This was the case for the basic mechanical parameters characterizing the whole LVPM, such as maximum shortening velocity (Vmax) (Figure 2A) and total muscle tension (Figure 2C). Other parameters characterizing the myosin CB molecular properties also decreased during anoxia, such as the CB single force (Figure 2B), the CB detachment rate constant g2 (Figure 3A), and the molecular content in myosin per g of tissue (Figure 2D). All these parameters diminished progressively and significantly during anoxia compared to their reference values before anoxia. These parameters are represented in Figure 2A–C and Figure 3A together with the molecular content in myosin per g of tissue (Figure 2D).

### 2.2. Coefficient of Friction, Frictional Force and Normal Load

Normal load (W) decreased during anoxia (Figure 3B). Other parameters increased during anoxia, such as the friction coefficient (Figure 3C) and frictional force (F) (Figure 3D).

### 2.3. Thermodynamic Force and Thermodynamic Flow

**Figure 4:**
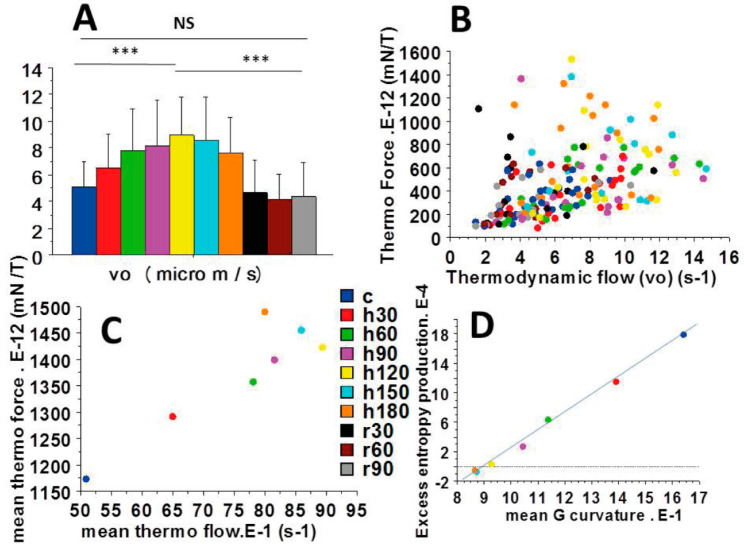
Non-linear thermodynamic force versus thermodynamic flow relationship. The myofilament sliding velocity (vo), or thermodynamic flow, is represented in (**A**); (**B**): Mean thermodynamic force versus mean thermodynamic flow; (**C**): Mean values of thermodynamic force versus thermodynamic flow (vo). ***: *p* < 0.001; (**D**): Linear relationship between EEP and the G curvature of the Hill hyperbola: EEP (10^−4^) = −22 + 2.4 G (10^−1^); r = 0.99.

The myofilament sliding velocity vo, or thermodynamic flow (Figure 4A), increased until reaching a maximum at about 120 min of anoxia and then decreased from 120 to 180 min of anoxia. Figure 4B,C show the absence of relationship between the thermodynamic force (F/T) and the thermodynamic flow (vo). Importantly, F/T varied non-linearly with vo. This means that the cardiac tribosystem operated in a non-linear fashion and thus far-from-equilibrium. In Figure 4B all values are presented, whereas mean values are shown in Figure 4C.

### 2.4. Partial Time Derivatives of Thermodynamic Force and Thermodynamic Flow

**Figure 5:**
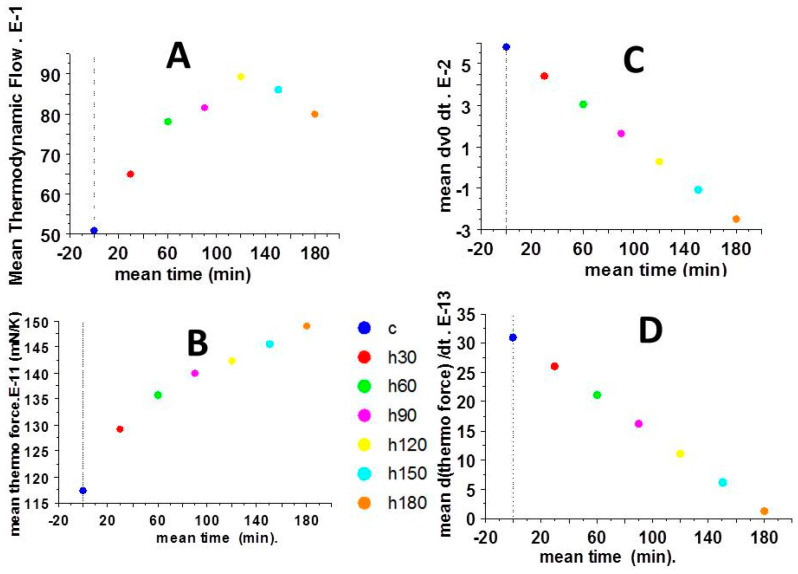
Mean thermodynamic parameters. (**A**): Mean thermodynamic flow versus time; (**B**): mean thermodynamic force versus time; (**C**): mean thermodynamic flow versus partial time derivative; (**D**): mean thermodynamic versus partial time derivative.

Figure 5 shows the mean values of the thermodynamic flow (vo) (Figure 5A) and thermodynamic force (F/T) (Figure 5B) versus time and their respective partial time derivative (Figure 5C,D). F/T increased over time and its partial time derivative ∂ (F/T) decreased but remained always positive (Figure 5D). Importantly, the partial time derivatives of vo, (∂vo) was positive up until 120 min, and then became negative from 150 to 180 min (Figure 5C).

### 2.5. Entropy Production Rate (EPR) and Excess Entropy Production (EEP)

**Figure 6:**
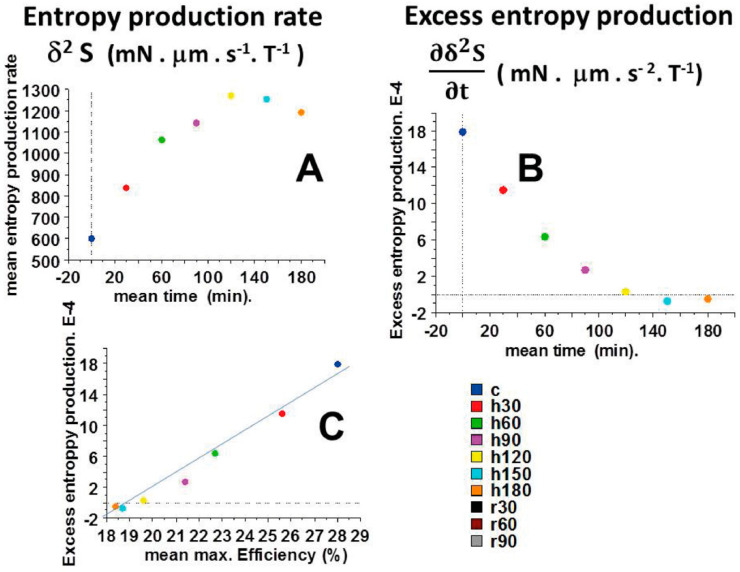
Entropy production rate (EPR) and excess entropy production (EEP). Entropy production rate (EPR) (in mN · μm · s^−1^ · T^−1^) versus time is represented in (**A**). (**B**) presented excess entropy production (EEP) (in mN · μm · s^−2^ · T^−1^ versus time. Note that EEP became negative from 150 to 180 min of anoxia. (**C**) showed the linear relationship between mean EEP and mean max. Efficiency (EEP = 1.9 max. Efficiency-3 7) (r = 0.90). EEP became negative when max. Efficiency was <19%.

Figure 6 shows the mean values of the entropy production rate (δ^2^ S) (Figure 6A) and the mean values of the excess entropy production ($\frac{\;\partial \delta^{2}S}{\partial t}\;$) versus time (Figure 6B). Up to 120 min of anoxia, EPR increased and then decreased from 120 to 180 min. EEP decreased continuously during anoxia but, importantly, remained positive up to 120 min of anoxia, after which it became negative (from 150 to 180 min of anoxia). The fact that EEP became negative after 150 min of anoxia demonstrates the instability of the non-linear cardiac tribosystem. Interestingly, there was a linear relationship between EEP and the CB detachment rate g2 (Figure 4D; EEP = 2.4 g2 − 22; r = 0.99). There was also a linear relationship between EEP and the maximum efficiency (Figure 6C) (EEP = 1.9 max. Efficiency-3 7; r = 0.90).

### 2.6. Self-Organization and Dissipative Structures

Self-organization can begin only if the system has lost its thermodynamic stability, which is characterized by the fact that EEP becomes negative. This is a pre-requisite for self-organization and this was the case in our study. Dissipative structures occur far-from-equilibrium under the following conditions: (i) the system must be open; (ii) it operates far-from-equilibrium; (iii) under a non-linear regime; (iv) and is submitted to *fluctuations*. Dissipative structures are maintained by thermodynamic processes which take place because of the exchange of energy between the system and its environment. They disappear as soon as that exchange ceases. All these conditions were observed in the studied cardiac system. The heart is an open system. Under continuous anoxia during 3h, it was submitted to slight fluctuations, it operated far-from-equilibrium due to the non-linearity between the thermodynamic force and the thermodynamic flow, and re-oxygenation induced a large reversibility of thermodynamic abnormalities.

A fundamental point is introduced by the variations of the sliding velocity (i.e., vo, the thermodynamic flow) (Figure 4A); vo increased, reached a maximum and then decreased during cardiac anoxia. Huxley’s equations show that vo is inversely proportional to the time stroke (ts) (Equation (7)). This means that, in the face of prolonged anoxia, the myosin head underwent ultrastructural changes, capable of modifying its molecular kinetics. Time stroke is a key step of the actin-myosin cycle. During this state, the myosin head generates a displacement of about 10 nm and a unitary CB force of some picoNewtons (Figure 2B). Both sliding velocity (vo) and time stroke (ts) returned to their control values after re-oxygenation. These changes in molecular kinetics of the myosin head due to changes in friction coefficient corresponded to changes in its molecular ultrastructure. This conferred to the myosin head a new ordered configuration that represents a dissipative structure during the anoxic period.

### 2.7. Re-Oxygenation

Re-oxygenation induced considerable reversibility of abnormalities of the LVPM mechanical indices due to anoxia. Reversibility was complete for several indices during re-oxygenation. This was the case for the maximum unloaded shortening velocity (Vmax) (Figure 2A), the myofilament sliding velocity (vo) (Figure 4A), the individual CB force (Figure 2B), the detachment rate constant g2 (Figure 3A), the frictional force (Figure 3D), and the friction coefficient (Figure 3C). Other indices, however, exhibited a partial reversibility and thus did not return completely to their control values. This was the case of the normal load (Figure 3B) and the molecular myosin content per volume unit of tissue (Figure 2D). Thus, anoxia induced a definitive CB loss of about 20%. Nevertheless, after re-oxygenation, the molecular characteristics of surviving CBs (i.e., po (Figure 2B) and g2 (Figure 3A) returned to the level of their respective control values.

## 3. Discussion

Prolonged myocardial anoxia induced dramatic abnormalities in molecular properties of the cardiac myosin. However, a high degree of reversibility of mechanical and thermodynamic defects was observed after re-oxygenation. A. Huxley has proposed the theory of sliding filaments, reflecting the friction of myosin filaments along actin filaments during contraction [1]. Based on this, we were able to apply the laws of tribology to the heart. A. Huxley also established a complex mathematical formalism [3] that made it possible to calculate the molecular mechanical properties of myosin CBs, including the force of a single CB, the maximum rate constant of CB detachment, the myosin content, and the sliding velocity of myofilaments. Myocardial anoxia resulted in a gradual decrease of mechanical indexes including the thermodynamic force (F/T). On the contrary, the myofilament sliding velocity (vo) increased up to 2 h of anoxia and then decreased from 2 h to 3 h of anoxia.

The entropy production rate (EPR) is the product of thermodynamic force (F/T) and thermodynamic flow (vo). We established that F/T and vo evolved in a non-linear fashion (Figure 4B,C). This demonstrated that the cardiac tribosystem operated far-from-equilibrium. Moreover, EEP became negative after 120 min of anoxia (Figure 6B), showing that the system became unstable. These results accounted for the occurrence of self-organization and dissipative structures in the anoxic heart. The stability condition for the tribological system is given by the expression of the second variation of the entropy production rate (δ^2^S > 0) (Equation (8)). Otherwise, the tribosystem became unstable and then a transition to a self-organized state could occur when δ^2^S < 0. In our study, this was the case after 150 min of anoxia (Figure 6A). Importantly, there was a linear relationship between EEP and max.Efficiency. Instability of the cardiac tribosystem occurred when max.Efficiency became <19% (Figure 6C).

The empirical Amontons–Coulomb law states that the frictional force varies linearly with the normal load (F = μW) (Figure 1E). Other linear empirical laws of physics, such as Fourier’s law of heat conduction, Ohm’s law of electrical conductivity, and Fick’s law of diffusion, link the thermodynamic flow to the thermodynamic force, the product of which represents the entropy production rate (EPR). The approximate Amontons–Coulomb law remains valid for numerous classes of materials: metals, ceramics, polymers, composites, etc. In tribology, several works on self-organization have been carried out over the past 40 years: [11,12,13,23,24,25]. Friction and wear are irreversible processes that lead to energy dissipation and material deterioration. Under certain circumstances, frictional sliding can result in the formation of spatial and temporal patterns, i.e., self-organization. This results from the tendency of energy and matter to achieve a complex disordered state that can lead to self-organization [10,13,24,26,27]. It has been proposed an entropic criterium for friction-induced self-organization [13,14,15,16,24].

In our study, the cardiac tribosystem revealed a fine adaptive process that provided a considerable resistance to anoxia. This process delayed cell death of cardiomyocytes and allowed them to remain contractile cells. This behavior was the result of a self-organization process. Several systems with dissipative structures and self-organization have been described in earlier studies, i.e., chemical oscillations, reaction of Belousov–Zhabotinsky, Brusselator, and Oregonator chemical models, Turing structures; turbulent liquid motion, Bénard cells, biomolecular asymmetry, etc. [10]. In tribology, an example of such a system is the beneficial lubricating action of tribofilms during machining [18]. In techniques of material synthesis (for example, physical vapor deposition), coating deposition favors mechanisms that occur far-from-equilibrium, leading to a high density of lattice imperfections [28] and creation of a surface with a highly non-equilibrium state, accelerating beneficial physicochemical reactions through the appearance of dissipative structures [18,19,24]. The occurrence of self-organization can be suggested if the wear rate is reduced [24]. In biological systems and beside friction force, numerous physical forces provide important non-chemical roles particularly during morphogenesis of embryos. Many examples can be mentioned such as gravity [29] and surface tension due to intercellular adhesion [30,31]. A decrease in entropy production rate, i.e., self-organization, is implied in these processes. Thermodynamics in life has been initially raised by Schrödinger [32].

After re-oxygenation, the thermodynamic abnormalities were found to be largely reversible, demonstrating that the deleterious effects induced by anoxia were not totally irreversible. Importantly, re-oxygenation after 3 h of anoxia induced a return towards a thermodynamic status relatively close to that of the initial state. However, two indices did not totally return to their control values, i.e., myosin content (Figure 2D) and normal load (Figure 3B). The occurrence of self-organization is ubiquitous in natural systems, particularly in biological complex open systems [10]. The fact that the myocardium reacted in this way after such prolonged anoxia bears witness to the incredible ability of nature to generate self-organization processes capable of resisting and delaying cardiac cell death. Self-organization probably prevented the death of a significant number of myosin heads, which would have otherwise irreversibly and dramatically impaired the contractile function of the cardiomyocytes after prolonged anoxia.

## 4. Methods

### 4.1. Ethical Statement

All experimental procedures conformed to the Guide for Care and Use of Laboratory Animals. The study protocol was approved by the Ethical Committee of the Institut National de la Santé et de la Recherche Médicale (INSERM), Paris, France. The research complied with the commonly accepted ‘3Rs’.

### 4.2. General Study Approach

Our study sought to determine EPR generated by the sliding of actin filaments along myosin filaments during the contraction phase (Figure 1A–E). EPR was the product of the thermodynamic frictional force (F/T) and the thermodynamic flow (vo); vo was the sliding rate of myofilament and was calculated from the A. Huxley equations [3]. Once EPR was determined, the next step was to calculate EEP and to see whether it turned negative after a prolonged period of anoxia, characterizing the instability of the cardiac tribosystem and the occurrence of self-organization [10,13,14,26].

### 4.3. Experimental Procedure

Experiments were conducted on 27 adult male rats from Charles River Laboratories. Rats were anesthetized with pentobarbital sodium (50 mg/kg ip). Left ventricular papillary muscles (LVPMs) were carefully dissected from the heart. Each LVPM was rapidly mounted in a tissue chamber containing a Krebs–Henseleit solution (in mM): 118 NaCl; 24 NaHCO_3_; 4.7 KCl; 1.2 MgSO_4_ 7H_2_O; 1.1 KH_2_PO_4_; 2.5 CaCl_2_ 6H_2_O; 4.5 glucose. The solution was bubbled with 95% O_2_-5% CO_2_ and maintained at pH 7.4 and 29 °C. LVPMs were electrically stimulated by means of two platinum electrodes delivering a 5ms stimulus at 0.1 Hz frequency. Experiments were carried out at Lo, i.e., the initial resting length corresponding to the apex of the length-active tension curve. The experimental procedure and the electromagnetic lever system have been previously described [33]. Maximum unloaded shortening velocity (V_max_, in Lo · s^−1^) of the LVPM was measured by means of the zero-load clamp technique [34]. Peak isometric tension (T, in mN/mm^2^; force per cross-section area) was measured from the fully isometric contraction. The tension-velocity relationship was derived from the peak velocity (V) of 8 to 10 isotonic afterloaded contractions, plotted against the isotonic tension level (iT) and by successive load increments from zero-load up to peak isometric tension (T) [33]. The tension-velocity (iT-V) relationship was fitted according to A.V. Hill’s equation (iT + a) (V + b) = [T + a] b where -a and -b are the asymptotes of the hyperbola [35]. For each LVPM, the iT-V relationship was accurately fitted by means of a hyperbola [33,35,36]. The curvature (G) of the hyperbola was T/a = Vmax/b.

### 4.4. A. Huxley’s Formalism

The use of the A. Huxley formalism [3] requires verifying that each LVPM presented a hyperbolic tension-velocity (iT-V) relationship. Values for the asymptotes -a and -b of the iT-V relationship were introduced into the Huxley equations. The rate of total energy release (E_Hux_) and isotonic tension (iT = F_Hux_) as a function of the LVPM velocity (V) were obtained by the following equations [3]:

E_Hux_ = (N e) (h/2 l) (f_1_/(f_1_ + g_1_)) {g_1_ + f_1_ (V/∅) [(1 − exp (−∅/V)]} (1)

F_Hux_ = N (w/2l) (f_1_/(f_1_ + g_1_)) {1 − (V/∅) [(1 − exp (-∅/V)) (1 + (1/2)) ((f_1_ + g_1_)/g_2_)^2^ (V/∅)]} (2)

where w was the maximum mechanical work generated by a single CB (w/e = 0.75) and e was the free energy required to split one ATP molecule. In the A. Huxley formalism, only one ATP was split per CB cycle. The standard free energy ΔG°′_ATP_ was −60 kJ/mol; e was equal to 10^−19^ J [37]. The myosin CB tilt relative to the actin filament varied from 0 to h; f_1_ was the maximum value of the rate constant for CB attachment; g_1_ and g_2_ were the maximum values of the rate constants for CB detachment; f_1_ and g_1_ corresponded to a tilt of the CB from 0 to h; g_2_ corresponded to a tilt > h; ∅ was equal to (f_1_ + g_1_) h/2 = b; and N was the number of CBs per cross-sectional area. The molecular step size h corresponded to the distance of translocation of the actin filament after the CB swing. The constant l was the distance between two successive actin sites with which a myosin head could bind. In agreement with the A. Huxley conditions (l >> h), h and l values were h = 10 nm and l = 28.6 nm. The parameters po, G curvature of the iF-V hyperbola, f_1_, g_1_, and g_2_ were calculated using the following equations:

G = f_1_/g_1_ (3)

g_1_ = 2wb/ehG (4)

g_2_ = 2Vmax/h (5)

po = (w/l) × [(f_1_)/(f_1_ + g_1_)] (6)

where po was the single CB force (in pN). Myosin content was calculated from the number of cycling myosin CB per volume unit of tissue and the Avogadro number. The sliding velocity of myosin filaments (in μm/s) along actin filaments was:

vo = h/ts(7)

where ts was the time stroke [38]. The rate of mechanical work (WM) is given by the formula: WM = P_HUX_ V. At any given load level, the mechanical efficiency (Eff) of the LVPM is defined as the ratio of WM to E_HUX_, i.e., Eff = WM/E_HUX_ and maximum efficiency (max. Efficiency) is the peak value of Efficiency.

### 4.5. Tribology and Heart Muscle

Friction is loosely defined as the resistance that a surface or object encounters when moving over another one. Bio-friction or “bio-tribology” can be defined as a friction applied to biological systems [39]. The principles of friction have been used for centuries in China and Egypt. Scientific studies on friction began with Leonardo da Vinci. Subsequently, Amontons and Coulomb laid the foundation for the current understanding of friction. Kinetic friction is an irreversible dissipative process. Friction represents the general tendency for irreversible energy dissipation in accordance with the Second Law of thermodynamics. The empirical Coulomb law of friction stipulates that the frictional force (F) is proportional to the normal load (W) according to the following formula (Figure 1E):

F = μW where μ the friction coefficient (without dimension) is independent of the normal load (W in mN), the myofilament sliding velocity (vo in μm/s), and the area of contact. The Coulomb frictional force (F in mN) is independent of the myofilament sliding velocity (vo).

The normal load (W) was calculated from Huxley’s equation. The myosin content per volume unit was calculated from Huxley’s equations [3] by dividing the CB number per volume unit by the Avogadro number. The myosin weight per volume unit was calculated knowing the myosin content and the myosin molecular weight. Cardiac myosin (molecular weight ~ 528 kDa) is a hexameric protein consisting of two myosin heavy chains (MHCs, 223 kDa), two pairs of regulatory light chains (RLCs, ~19 kDa), and two pairs of essential light chains (ELCs, ~22 kDa) [40]. The normal load W was determined by applying Newton’s second law, i.e., W = 9.81 × myosin molecular weight.

The coefficient of friction μ has been determined in numerous living tissues [41]. In a myosin filament sliding along an actin filament, μ has been determined to be equal to ~0.001 [42]. The higher the detachment rate constant g2, the easier the sliding of myosin along actin and the lower the friction coefficient μ. The coefficient of friction was equal to 0.001 × (g2 control/g2 anoxia). The thermodynamic flow (vo) was equal to the myofilament sliding velocity and the thermodynamic force was equal to the frictional force F divided by T (T: Kelvin temperature): thermodynamic force = μ W/T.

### 4.6. Stability Conditions

Thermodynamic variables can be considered as functions of time and position. This is the assumption for assuming the local equilibrium principle. The fluctuations induced by the anoxia were small, and mechanical and thermodynamic changes were very progressive and continuous during 3-h.

In a non-equilibrium system and in the case of friction phenomena, the entropy production rate (EPR) is given by the expression:

δ^2^ S = (1/T)·F·v0, (in mN·μm·s^−1^·T^−1^) (8)

where S is the entropy of the tribosystem. The transition from a stationary state sliding regime to a regime with self-organization occurred through the destabilization of the stationary state. Equation 8 is considered as a Lyapunov function. The second variation of entropy (δ^2^ S) corresponds to the entropy production rate (EPR) which relates to a disturbance of the system due to anoxia. The stability condition for the thermodynamic system is given by the following equation:

(9) $$\frac{\partial \delta^{2}S}{\partial t}=\frac{\partial F}{T}\delta \mathrm{vo}\;>\;0$$

$\frac{\partial \delta^{2}S}{\partial t}$ is the excess entropy production (EEP).

The system is unstable when the value obtained through Equation (9) is negative.

Here (1/T) δF and δvo are the deviations of the thermodynamic force (F/T) and the thermodynamic flow (vo) from the stationary state, respectively. If EEP remains positive, the system remains stable. When the deviation from the stationary state goes beyond a certain critical value, the system is then able to reach an instability threshold. At a given value, EEP may change its sign, may become negative and thus unstable, and a transition to a self-organized solution with patterns can occur. A criterion based on the loss of thermodynamic stability (EEP < 0) is an indication of the occurrence of self-organization [4,9,10,18,19]. The necessary condition for the occurrence of self-organization is EEP < 0.

### 4.7. Statistical Analysis

Data were expressed as means ± standard deviations. Comparisons of parameters between groups were performed using Student’s unpaired *t*-test. A *p* value < 0.05 was considered statistically significant.

## 5. Conclusions

Friction is a process based on the concepts of non-equilibrium thermodynamics and self-organization. Within a tribosystem, self-organization may lead to dissipative structures that occur in far-from-equilibrium open systems, resulting in a decrease in wear rate [24]. The fundamental concepts of non-equilibrium thermodynamics, entropy, self-organization, dissipative structures, and thermodynamic stability-instability analysis represent powerful tools that have been used to develop innovative materials with substantial benefits. In our study, during prolonged anoxia, the heart was an open non-linear thermodynamic tribosystem operating far-from-equilibrium, as asserted by the non-linearity between the thermodynamic force and the thermodynamic flow. The negative value of the excess entropy production after 2 h of cardiac anoxia enabled self-organization. Anoxia led to dramatic changes in the friction coefficient, inducing modifications to CB kinetics which are largely reversible after re-oxygenation Such an adaptation was related to the occurrence of dissipative structures [24]. This anoxic cardiac tribosystem was thus able to shift from a severe wear mode to a milder one. These results are of particular interest in the context of heart transplants where the time that elapses between heart removal from the donor and its transplantation in the recipient is crucial. Self-organization and dissipative structures appear to prevent, or at least to limit, the appearance of dramatic irreversible damage to the myosin CBs.

## Data Availability

Not applicable.
